# Assessing causality by means of the Naranjo scale in a paediatric patient with life threatening respiratory failure after alemtuzumab administration: a case report

**DOI:** 10.1186/s12887-021-02698-w

**Published:** 2021-05-12

**Authors:** Nori J. L. Smeets, Ruud J. R. Eijk, Saskia N. de Wildt, Charlotte M. H. H. T. Bootsma-Robroeks

**Affiliations:** 1 Department of Pharmacology and Toxicology, Radboud Institute of Health Sciences, Radboud University Medical Center, 6525 EZ Nijmegen, The Netherlands; 2 Department of Intensive Care and Pediatric Surgery, Erasmus MC—Sophia Children’s Hospital, 3015 GJ Rotterdam, The Netherlands; 3 Department of Pediatric Intensive Care, Radboud University Medical Centre, 6500 HB Nijmegen, The Netherlands; 4 Department of Paediatric Nephrology, Radboud University Medical Center, Amalia Children’s Hospital, Nijmegen, The Netherlands

**Keywords:** Alemtuzumab, Diffuse alveolar oedema, Naranjo scale, Respiratory failure, Case report

## Abstract

**Background:**

Alemtuzumab is a T cell depleting antibody agent used as induction immunosuppressant therapy in solid organ transplant recipients. In addition, it is being increasingly used to treat severe or glucocorticoid-resistant graft rejection. Despite the effectiveness of the treatment, severe adverse events have been reported related to alemtuzumab administration. We present a similar event illustrating the severity of this adverse drug reaction (ADR) and we highlight the structure causality assessment provides in approaching such a case.

**Case presentation:**

We report a case of life-threatening respiratory failure after alemtuzumab administration in a 17 year old paediatric kidney transplant recipient. He developed near fatal severe respiratory and circulatory failure based on acute respiratory distress syndrome (ARDS) with diffuse alveolar oedema and haemoptysis hours after his second alemtuzumab administration. As it was questionable whether alemtuzumab could be regarded as the origin of his reaction and in order to assess the causality of this reaction as well as to structure clinical reasoning, we applied a widely used ADR probability scale to systematically review our case.

**Discussion and conclusions:**

Our case shows a severe ADR after alemtuzumab administration. It illustrates the importance of proper causality assessment, the structure it provides and the benefit of a clinical pharmacology consultation when a severe reaction is suspected to be an ADR. By taking our case as an example, we demonstrate the added value of structured causality assessment to clinical reasoning and in generating differential diagnoses.

## Background

Paediatric kidney transplantation is the treatment of choice for patients with end-stage renal disease (ESRD). It significantly improves survival, growth and health-related quality of life compared to dialysis [1, 2]. In the last decades, overall graft survival has significantly improved, among others due to changes in both type of immunosuppressive agents and regimens [3, 4]. Alemtuzumabs mechanisms of action are CD52 antibody dependent cellular cytolysis and complement-mediated lysis following binding to these cells. CD52 is a peptide of 12 amino acids, anchored to glycosylphosphatidylinositol. On the cell surface of T and B lymphocytes, high levels of CD52 peptide are present thus leading to T and B cell depletion. Alemtuzumab is approved as a single disease modifying therapy in adults with highly active relapsing remitting multiple sclerosis (MS) [5]. In solid organ transplantation, alemtuzumab is increasingly being used as an induction immunosuppressant agent and for treating acute glucocorticoid-resistant rejection as alternative for rabbit anti-thymocyte globulin (rATG) therapy [6]. While evidence supports similar efficacy and a better safety profile compared to rATG, post-marketing pharmacovigilance shows rare but serious side effects including cardiovascular and immune-related disorders [6, 7]. We describe a 17-year old patient who developed near fatal severe respiratory and circulatory failure based on acute respiratory distress syndrome (ARDS) with diffuse alveolar oedema and haemoptysis shortly after the second alemtuzumab subcutaneous dose. Although previous case reports have described similar severe symptoms in relation to alemtuzumab, we demonstrate the importance of proper causality assessment and the benefit of a clinical pharmacology consultation. We apply a widely used adverse drug reaction (ADR) probability scale as developed by Naranjo to systematically assess causality [8]. By doing so, we demonstrate the added value of this structured approach to clinical reasoning and in generating differential diagnoses.

## Case presentation

A 17-year old boy with a history of autosomal recessive polycystic kidney disease (ARPKD) started haemodialysis when he was 4 years of age. At the age of 8 years, he received a diseased donor kidney transplant. After 8 years he lost this graft due to chronic transplant rejection and haemodialysis needed to be restarted. Two years later, a second kidney transplant was executed by means of a heart beating donor. Notably, this donor had a repeated HLA mismatch of B14. However, levels of donor specific antibodies against B14 were not detected in his last pre-transplant serum sample.

According to our local guideline, induction after transplantation was initiated with basiliximab. Tacrolimus, mycophenolate mofetil and prednisolone were given as maintenance immunosuppression therapy. Because of altered graft function (creatinine 394 μmol/L and oliguria (0.3–0.8 ml/kg.h), unresponsive to furosemide), tacrolimus was discontinued 4 days post transplantation. Day 11 post-transplant, graft function deteriorated even further (creatinine 415 μmol/L, blood urea nitrogen 36 mmol/L). After a renal biopsy was performed, methylprednisolone pulses were initiated and low-dose tacrolimus was restarted. In addition, on day 12, plasmapheresis was started for five consecutive days in order to treat a possible humoral rejection. The graft biopsy demonstrated a type 2A acute rejection (BANFF classification: C4d positive, diffuse interstitial infiltrate, extensive oedema, focal interstitial hemorrhage, mild tubulitis, mild acute glomerulopathy and mild endovasculitis) [9]. Polyoma virus in plasma was negative, and no viral inclusions were found in the biopsy. Due to his underlying ARPKD, leucopenia and thrombocytopenia were present.

**Fig. 1:**
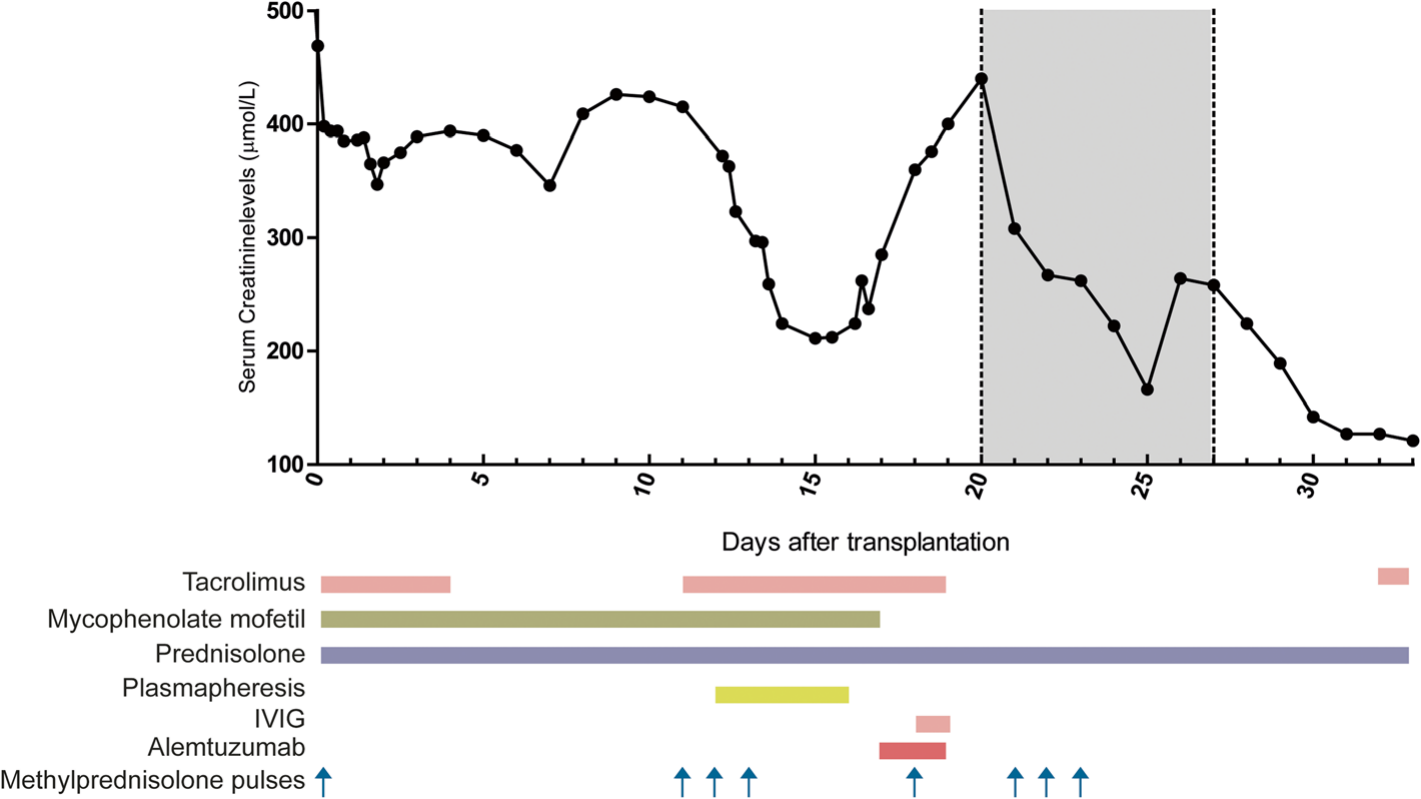
Serum creatinine levels during clinical course in μmol/L and administered drugs. Grey area reflects admission at PICU

Donor-specific human leukocyte antigen (HLA) antibodies against B14 in plasma increased significantly. Creatinine initially decreased from 425 μmol/L to 215 μmol/L but increased again to 376 μmol/L on day 18 (Fig. 1). Therefore, intravenous immunoglobulin G (IVIG) was administered. Graft function improved and tacrolimus was doubled at day 15 to a dose of 1 mg (0.02 mg/kg).

A second graft biopsy, on day 18 post-transplant, showed severe cellular rejection. There were minor signs of humoral rejection as a mild influx of inflammatory cells in the capillary lumen was observed and donor specific antibodies (DSA) against B14 mildly increased. In order to treat this severe steroid resistant cellular rejection, with a mild humoral component in the biopsy and mild DSA against B14, anti T-cell therapy was indicated.

A splenectomy as well as rituximab therapy were both considered but not deemed suitable. Splenectomy to treat antibody-mediated rejection (AMR) remains debatable as antibodies are not solitary being produced by the spleen, but also in lymph nodes and bone marrow. This, combined with the increased risk of infection with capsuled bacteria, argued against splenectomy. Rituximab on the other hand, targets CD20, mainly present on memory B-cells. However, plasma-cells responsible for antibody production lead to CD138 expression and do not express CD20. The leuco- and thrombocytopenia present in this patient resulted in the choice to treat this patient with alemtuzumab instead of rATG, as less side effects were expected compared to other anti-T-cell therapies.

Alemtuzumab was administered subcutaneously at a dose of 30 mg. One hour prior to administration, 500 mg methylprednisone was given together with 2 mg clemastin to prevent allergic reactions and 500 mg acetaminophen to prevent development of fever and/or headaches. In order to prevent raised levels of uric acid due to a tumour lysis syndrome, 200 mg allopurinol was given as a prophylaxis and hyper hydration protocol was applied (1,5–2,0 L/m^2^ per 24 h). Except for a minor headache 4 h post administration, no side effects occurred. Two days later, tacrolimus was discontinued due to high potassium levels and the second dose of alemtuzumab was administered.

Seven hours after this second dose, patient complained about a headache similar to the day before and he developed a fever up to 38.5 degrees Celsius. Within 5 h, his clinical status deteriorated significantly. The patient became dyspnoeic (oxygen saturation 87%) with 3 L of 100% oxygen and he started coughing. He was in agony, tachypnoeic (45–50/min) with a 15 L non rebreathing mask and his heartrate increased to 150/min with a mean arterial pressure of 95 mmHg.

Furosemide 80 mg was administered intravenously without effect and the patient was transferred to the paediatric intensive care unit (PICU) and immediately intubated due to acute respiratory failure. He deteriorated within hours into severe acute respiratory distress syndrome (ARDS) with signs of pulmonary oedema (foamy bloody secretions). Mechanical ventilation with maximal settings with nitric oxide (20 ppm) and abdominal positioning as well as maximal inotropic treatment with adrenalin (max 0,7 μg/kg/min), vasopressin (max 0,04 IE/min), dobutamine (max 33 μg/kg/min) and noradrenalin (max 2,1 μg/kg/min) were required. Ascites (3 L) was drained to lower ventilatory pressure before transferring the patient into supine position. Cardiac ultrasound showed diminished hypertrophic left ventricle contractility without signs of backward failure, a normal right ventricle and a collapsed inferior vena cava. There were no signs of fluid overload. Fluid resuscitation with crystalloids, packed cells and thrombocytes was initiated. Extracorporeal membrane oxygenation was not possible due to lack of appropriate vascular access. Continuous Renal Replacement Therapy (CRRT) was started 6 h after admission to the PICU as acute kidney injury and general oedema developed due to capillary leak syndrome. Yet, this failed to improve his clinical status. As opportunistic infections as a cause for the deterioration could not be excluded, antibiotics, ganciclovir and fluconazole were given. Unfortunately, the patient was too unstable to perform a broncho-alveolar lavage for culture diagnostics. Continuing this maximal supportive therapy, the patient stabilized after 12 h and inotropic and vasopressive support could be tapered and ventilation and oxygenation gradually improved. After 5 days on mechanical ventilation, he was successfully extubated and could be discharged from the PICU to the ward after an additional 8 days. He returned home in relatively good condition 33 days after his kidney transplantation with a serum creatinine of 111 μmol/L.

## Discussion and conclusions

This report shows a case of a severe respiratory and circulatory failure based on ARDS with diffuse pulmonary oedema and haemoptysis. Uncertainty remained with regards to the cause of this unexpected, sudden and extremely severe deterioration. With a high likelihood of an alemtuzumab ADR, a clinical pharmacology consult was requested and a structured approach was conducted by using the Naranjo ADR probability score. Although organ-specific scores exist and have a higher validity [10], the Naranjo scale remains one of the most accessible and therefore most frequently used ADR probability scale in case of a single-drug-event [11]. We therefore used this scale and demonstrate that, by applying this simple tool and asking predefined questions, proper causality assessment leads to better substantiated clinical reasoning and might help in generating differential diagnoses. This could therefore be considered as a first and accessible step for any clinician encountering a possible ADR.

Naranjo et al. developed the ADR Probability Scale in 1991 in order to determine the likelihood of an ADR being actually due to administration of a drug rather than the result of other factors [8]. By addressing the items of the scale (Table 1), we will systematically discuss our case and assess the probability of alemtuzumab as the cause of the acute ARDS.

**Table 1:** Adverse drug reaction probability scale by Naranjo

	Yes	No	Unknown
1. Are there previous conclusive reports on this reaction?	+ 1	0	0
2. Did the adverse event appear after the suspected drug was administered?	+ 2	−1	0
3. Did the adverse event improve when the drug was discontinued or a specific antagonist was administered?	+ 1	0	0
4. Did the adverse reaction reappear when the drug was readministered?	+ 2	−1	0
5. Are there alternative causes (other than the drug) that could on their own have caused the reaction?	−1	+ 2	0
6. Did the reaction reappear when a placebo was given?	−1	+ 1	0
7. Was the drug detected in the blood (or other fluids) in concentrations known to be toxic?	+ 1	0	0
8. Was the reaction more severe when the dose was increased, or less severe when the dose was decreased?	+ 1	0	0
9. Did the patient have a similar reaction to the same or similar drugs in any previous exposure?	+ 1	0	0
10. Was the adverse event confirmed by any objective evidence?	+ 1	0	0

There are previous reports on similar reactions after alemtuzumab administration (point 1). Six articles describe pulmonary reactions [12–17] including eight patients between 16 and 54 years of age. In these patients, alemtuzumab was prescribed for relapsing remitting MS (*n* = 6) or induction after kidney transplantation (*n* = 2).

The severity and onset of the ADR after alemtuzumab differed greatly between patients. All patients experienced dyspnoea, fatigue and coughing, but only three patients required ICU admission for respiratory insufficiency [15–17] of which one patient died [16]. Symptoms started between 24 h after the first administration [15] to up to 2 months after the first five-day cycle [12]. In our patient, respiratory failure occurred 7 h after the second dose and quickly worsened (point 2).

Other causes for ARDS in our patient were extensively reviewed and were considered less likely (point 5). Fluid overload related pulmonary oedema was rejected as administration of diuretics nor CRRT ultrafiltration improved the ARDS. Also, an infectious cause was unlikely with an only moderately increased C-reactive protein (CRP) (maximum of 62 mg/L, 24 h after the second administration) and blood cultures did not show any growth. Thrombocytopenia related fatal alveolar haemorrhage was also less likely with a thrombocyte count of 34*10^9^/L.

Due to the severe clinical course of our patient, many answers of the Naranjo scale will remain unanswered. Dechallenge or changing the dose was not possible due to the very long half-life of alemtuzumab and rechallenge was obviously not tested in our patient (point 3&4). No placebo was administered and no plasma alemtuzumab concentration was measured (point 6&7). Our patient never experienced a similar reaction to a different drug before (point 9).

Last, diagnosis of ARDS with diffuse alveolar oedema and haemoptysis was supported based on bedside chest X-ray and the clinical status of the patient (point 10). This included chest imaging findings of new infiltrates consistent with acute pulmonary parenchymal disease as well as the need of mechanical ventilation with maximal support in abdominal positioning, combined with maximal nitric oxide, inotropic agents and CRRT. Using the paediatric ARDS criteria, this is considered severe paediatric ARDS (PARDS) as his respiratory failure could not be explained by cardiac failure nor by fluid overload [18].

The score of the Naranjo algorithm ranges between − 4 and 13 with 0 being a doubtful ADR and a score of 9 or higher is considered as a definite ADR [8]. Our assessment resulted in a score of 6 corresponding with a probable ADR. Although severe ADRs are not excluded from the assessment using the Naranjo probability scale, severe ADRs will have a lower total achievable score on an individual case basis as some items cannot be scored positive. For instance, for severe ADRs, a rechallenge will often not be feasible for safety reasons. Hence, the assessment can only result in possible or probable causality score but will never be considered as definitive (final score ≥ 9). In our case, this needs caution to interpret the final score and to dismiss this as only a probable ADR without taking the severity into account.

These shortcomings of the Naranjo scale were also addressed by García-Cortés et al. in cases of suspected hepatotoxicity [10]. It was demonstrated that the Naranjo scale lacks validity and reproducibility in the attribution of causality in hepatotoxicity and is considered less suitable when organ-specific causality scales are available. However, we are not aware of a scale specifically designed to assess causality in case of respiratory failure and used the Naranjo scale to demonstrate its value in structurally reviewing our case. Many other causality assessment methods of ADRs are available, each with their own criteria for assigning causality and an excellent overview was given by Agbabiaka et al. [11]. Some methods exclude items on rechallenge and/or the dose-effect relation and, therefore, might be better for severe ADR causality assessment. However, the strength of any ADR probability scale is that it provides simple tools for systematic appraisal in assessing the causality of any ADR. Using these tools therefore leads to a more structured clinical reasoning that might be of help when generating differential diagnoses. In addition, besides this assessment, we plea to be aware of this rare but very serious ADR of alemtuzumab, especially in kidney transplant recipients where rejection is increasingly being treated with this agent.

## Data Availability

Not applicable.

## Abbreviations

ADR: Adverse drug reaction
ARDS: Acute respiratory distress syndrome
ARPKD: Autosomal recessive polycystic kidney disease
AMR: Antibody-mediated rejection
ATG: Anti-thymocyte globulin
CRP: C-reactive protein
CRRT: Continuous renal replacement therapy
DSA: Donor specific antibodies
ESRD: End-stage renal disease
IVIG: Intravenous immunoglobulin G
HLA: Human leukocyte antigen
MS: Multiple sclerosis
PICU: Paediatric intensive care unit
